# Enhancing the Australian Gridded Climate Dataset rainfall analysis using satellite data

**DOI:** 10.1038/s41598-022-25255-6

**Published:** 2022-11-30

**Authors:** Zhi-Weng Chua, Alex Evans, Yuriy Kuleshov, Andrew Watkins, Suelynn Choy, Chayn Sun

**Affiliations:** 1 Bureau of Meteorology, Melbourne, VIC Australia; 2 Royal Melbourne Institute of Technology, Melbourne, VIC Australia

**Keywords:** Climate sciences, Hydrology

## Abstract

Rainfall estimation over large areas is important for a thorough understanding of water availability, influencing societal decision-making, as well as being an input for scientific models. Traditionally, Australia utilizes a gauge-based analysis for rainfall estimation, but its performance can be severely limited over regions with low gauge density such as central parts of the continent. At the Australian Bureau of Meteorology, the current operational monthly rainfall component of the Australian Gridded Climate Dataset (AGCD) makes use of statistical interpolation (SI), also known as optimal interpolation (OI) to form an analysis from a background field of station climatology. In this study, satellite observations of rainfall were used as the background field instead of station climatology to produce improved monthly rainfall analyses. The performance of these monthly datasets was evaluated over the Australian domain from 2001 to 2020. Evaluated over the entire national domain, the satellite-based SI datasets had similar to slightly better performance than the station climatology-based SI datasets with some individual months being more realistically represented by the satellite-SI datasets. However, over gauge-sparse regions, there was a clear increase in performance. For a representative sub-domain, the Kling-Gupta Efficiency (KGE) value increased by + 8% (+ 12%) during the dry (wet) season. This study is an important step in enhancing operational rainfall analysis over Australia.

## Introduction

Rainfall over Australia is highly variable in both space and time, more so than in similar climates around the world^1^. Associated with this is a vulnerability to hydrological hazards such as drought and flooding. The ability to uniformly assess rainfall over large areas is essential for monitoring onset, evolution, cessation and impacts of such hazards^2,3^. Being able to properly place drought and flooding into a historical perspective is also important for understanding past severity^4^ as well as projections^5^ of these climate extremes. Gridded rainfall datasets are invaluable in these climatological aspects, in addition to their usefulness in calibrating and verifying other datasets, including climate model output^6^.

Gridded analyses are commonly divided into three categories: gauge-based, satellite-derived, and model reanalyses. Gauge-based datasets rely on interpolation of point gauge data to a gridded form. Traditionally, this has been the only method used to monitor rainfall operationally at the Australian Bureau of Meteorology (BOM). In the early 2000s, an inverse-distance weighting scheme known as Barnes analysis was used^7^. Barnes analysis relies on forming an estimate based on the inverse exponential ratio of the distance between an observation and the grid cell, and a parameter related to the spacing of the observations^8^. Additional corrections known as 'passes' can be made by optimizing the residuals at the observational points. This dataset was superseded by the Australian Water Availability Project (AWAP) dataset in 2009 which introduced the use of an anomaly approach to the analysis^9^. An interpolation of anomalies via a multi-pass Barnes analysis was added to a climatology field derived using a thin plate spline (TPS). The TPS technique works by fitting a smooth surface to a set of data points^10^. The current operational dataset used at the BOM is the Australian Gridded Climate Dataset (AGCD). It was developed in 2019 and employs a method known as statistical interpolation (SI), also known as optimal interpolation (OI). Instead of superimposing an anomaly field onto a climatological background field like AWAP, SI relies on assimilating point observations onto a background field, also known as the 'first-guess', weighted according to information based on a priori estimates of the background and observational errors, as well as on the spatial correlation of the observations. The weights are determined through the minimization of the error variance of the resultant analysis to both the background field and the point observations.

**Figure 1:**
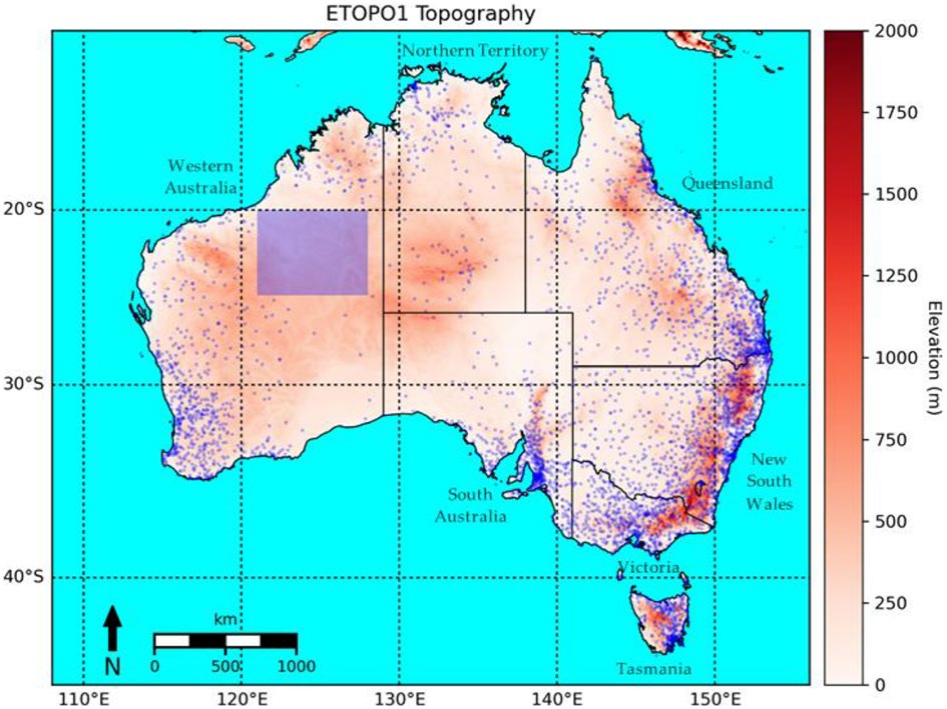
Typical rain gauge network coverage in Australia. The blue dots represent stations while the shading represents topography derived from the ETOPO1 dataset. The main topographical feature of the continent, the Great Dividing Range, can be seen running along the east coast of the continent, extending from northern Queensland to central Victoria. The blue rectangle represents a sub-domain used for validation of a gauge-sparse region. Figure created using Matplotlib 3.3.3 on Python.

In AGCD, the point observations are station observations from the BOM and the background field is the same climatology field used in AWAP, i.e. one derived from stations using TPS. A typical representation of the rain gauge network in Australia which consists of more than 6000 stations, along with the domain of this study is shown in Fig. 1, adapted from Ref.^11^. Topography was derived from the ETOPO1 dataset which is a National Oceanic and Atmospheric Administration (NOAA) model of land topography and ocean bathymetry formed from survey and remotely-sensed data, provided at a resolution of 1 arc-minute^12^.

The use of relative accuracies for weights means that where they exist, the analysis will lean heavily towards the values of stations but in data-sparse regions, the analysis will be closer to the 'first-guess', i.e. the climatological field. This is not optimal given the variability of rainfall, especially in Australia. Hence, not unexpectedly, the cross-validated root mean squared error (RMSE) normalised by the mean is the highest in the gauge-sparse regions of Australia^13^.

Expanding the gauge network in these remote regions is not economically feasible with a large number of stations required to resolve the paucity^7,13^. In addition to installation costs, these areas can be some distance from large population centers and hence would require high maintenance costs. While there may be a low requirement for local observations, these regions form a large part of the present analysis and are necessary for understanding water availability on a national scale.

A more practical way to improve the analysis would be the use of a more accurate 'first-guess' than station climatology. Remotely sensed precipitation estimates from satellites are an attractive prospect given satellites are able to generate a uniform representation of rainfall over most of the globe, including Australia. As an alternative data source available over a large domain, satellites have demonstrated they are a superior data source for non-polar latitudes and regions not covered by mountains^14,15^. This is appropriate for Australia, a region that encompasses the mid-latitudes and tropics, as well as having little significant mountainous terrain apart from the Great Dividing Range along the east coast of the continent. Accuracy of monthly satellite estimates of rainfall over Australia has been shown to be very reasonable^11^, thereby offering a strong potential for improvement over station climatology.

Operational BOM rainfall analyses have always only relied on gauge data, and so the use of alternative data sources such as satellites is a significant step in potentially improving the quality of the analysis and the intelligence derived from it. The objective of this study is therefore to explore the potential improvement in the monthly version of AGCD by using satellite data as a background field with the aim of producing an improved analysis. An improved analysis would provide a more accurate assessment of water availability across the region, facilitating better management of water-availability-related risks.

Both uncorrected and gauge-corrected Global Satellite Mapping of Precipitation (GSMaP) datasets were processed into a form that could replace the station climatology field used in AGCD. The AGCD process was then adapted to handle this field. The resulting datasets were evaluated, including a comparison against its station climatology counterparts. The next sections present the results and a discussion of the key findings. Readers are referred to “Methodology” for detailed explanations on the methodology and the datasets used.

## Results

### In-situ validation

This validation was based on the cross-validation statistics produced from the SI process and so only SI datasets were included. These statistics were formed by excluding every station one-by-one and then calculating an error for a station by comparing it to an intermediate analysis formed from its exclusion (i.e. a form of split-sample validation). For this study period, the number of stations available for use in SI (and in this validation) varied between 2712 and 6262, with a median of 5707. This is primarily due to historical changes in station availability (e.g., due to station closures or new installations) as well as delays in reporting time meaning less stations were available towards the end of the study period. A monthly error value was then obtained from the average error of all the stations available for that month.

**Figure 2:**
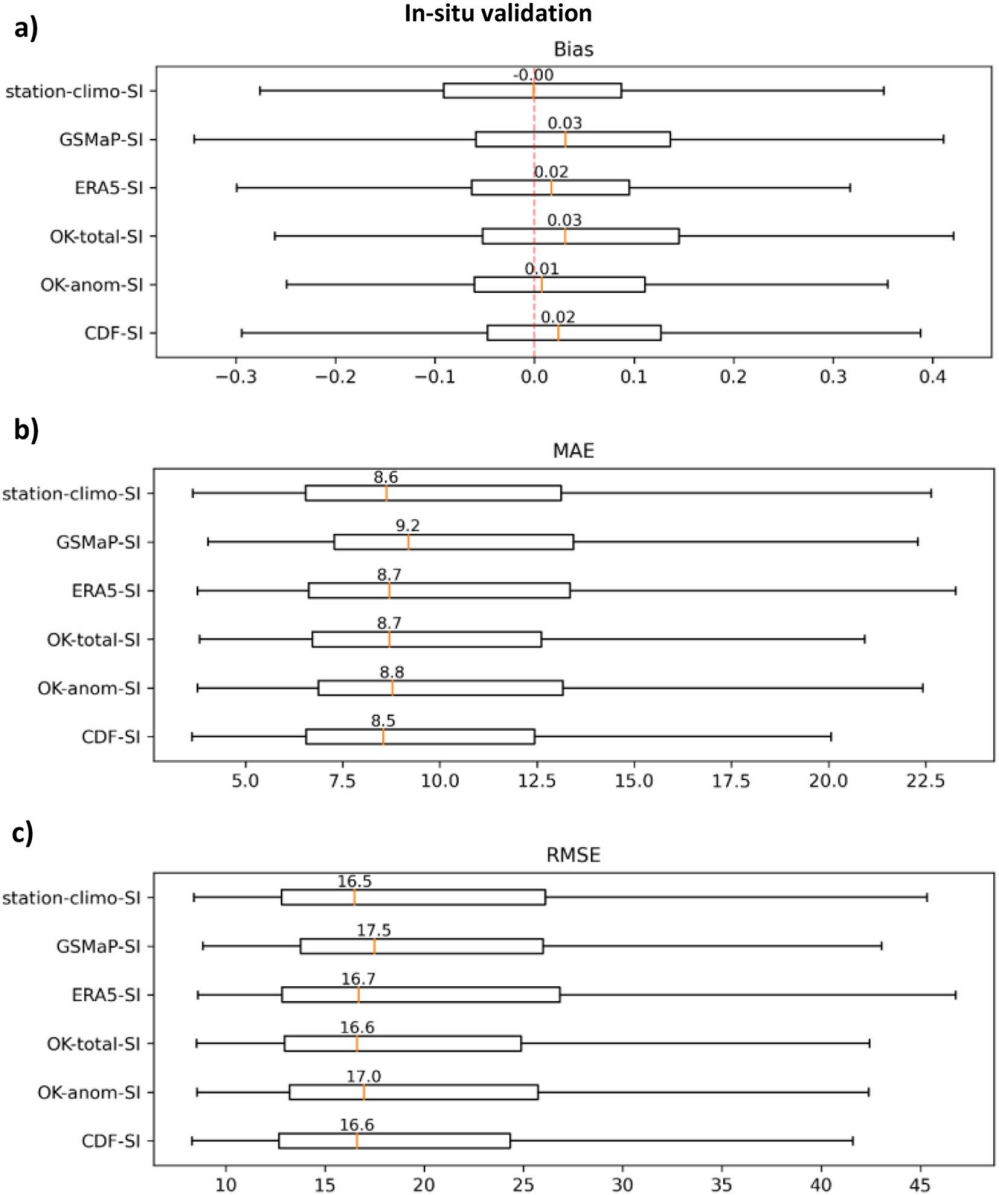
Boxplots of (**a**) bias, (**b**) root-mean-squared error (RMSE) and (**c**) mean absolute error (MAE) from in-situ cross-validation. Units are in mm/month. A value of zero is ideal for all three metrics. The boxes indicate the interquartile range (IQR), the whiskers extend out to the non-outlier minimum and maximums (Q1 − 1.5 × IQR and Q3 + 1.5 × IQR), and the line within the box represents the median.

These monthly errors are summarized in Fig. 2 (the corresponding time series are included in Appendix A). The prefixes of the datasets denote the background field used—station-climo-SI used station climatology, GSMaP-SI used uncorrected GSMaP, ERA5-SI used the ERA5 reanalysis, and OK-total-SI, OK-anom-SI and CDF-SI used a corrected version of GSMaP. Readers are referred to “Methodology” for a full explanation on the datasets used in this study.

**Figure 3:**
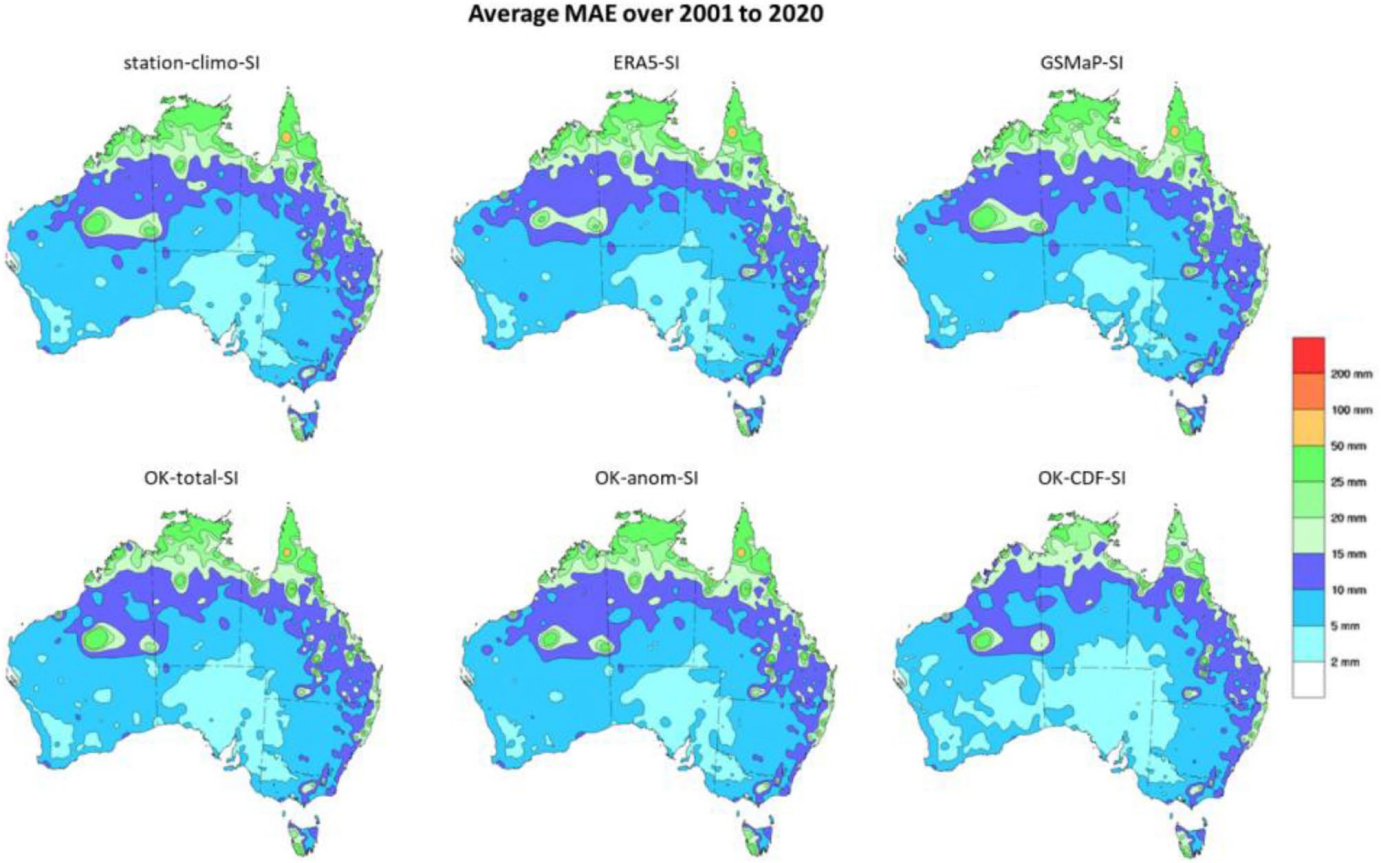
Spatial representation of the cross-validated in-situ MAE of the datasets over the period of 2001 to 2020. Units are in mm/month. Figure created using NCAR Graphics from NCL 6.1.2.

Using the uncorrected GSMaP (GSMaP-SI) yielded the worst results across all metrics, while the most performant dataset varied depending on the metrics used with the remaining datasets having similar performances. In terms of the MAE, CDF-SI had the lowest value, outperforming station-climo-SI. However, station-climo-SI had the lowest RMSE. OK-total-SI had slightly worse metrics, followed by OK-anom-SI. The distribution of errors for CDF-SI appeared to be the tightest amongst the datasets, with the bulk of the distribution being the smallest as well. The average MAE for the study period was calculated and shown in Fig. 3.

The spatial representations were similar displaying a general north–south MAE gradient, with higher values towards the north of the continent. In general, the satellite-SI datasets showed a slight reduction in the MAE over the northern interior of Western Australia compared to the station-climo-SI dataset.

### Multi-source weighted-ensemble precipitation (MSWEP) validation

**Figure 4:**
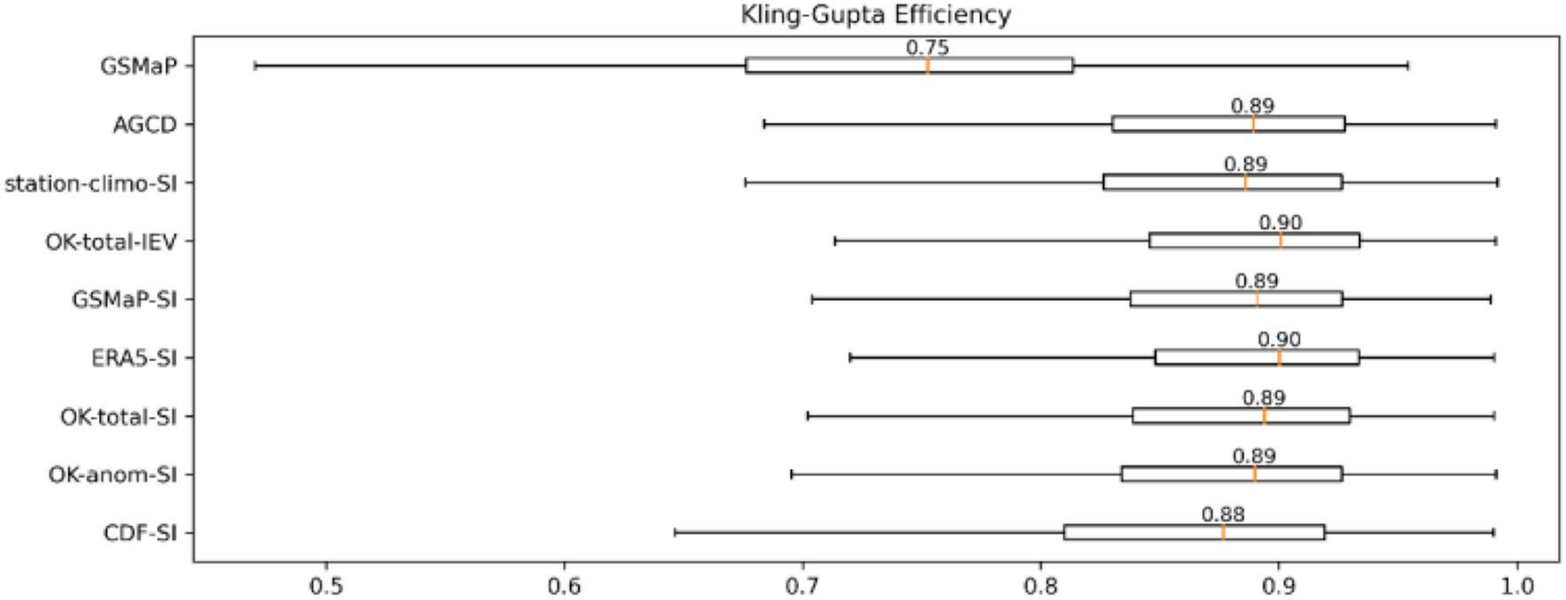
Boxplot of Kling-Gupta Efficiency (KGE) from MSWEP validation. A value of unity is ideal. The boxes indicate the interquartile range (IQR), the whiskers extend out to the non-outlier minimum and maximums (Q1 − 1.5 × IQR and Q3 + 1.5 × IQR), and the line within the box represents the median.

The results of the gridded validation against MSWEP (Multi-Source Weighted-Ensemble Precipitation) are shown in Fig. 4. OK-total-IEV is a gauge-satellite blended dataset we created in a previous study^16^. It was included as an additional reference as it performed the best in that study. Again, readers are referred to “Methodology” for details on these datasets. Only the boxplot for the Modified Kling-Gupta Efficiency (KGE) is presented for brevity, boxplots of its components (the bias ratio, variability ratio and Pearson correlation) are provided in Appendix B.

The SI datasets that use a background field based on satellite estimates (hereafter referred to as satellite SI datasets) performed similar or slightly better than their station climatology variants, apart from CDF-SI. The nuance of this is discussed in Sub-section Value of SI. ERA5-SI performed the best, with the blended satellite datasets lagging slightly behind. Note that ERA5 is an input into MSWEP and thus its performance was likely inflated; its inclusion was primarily as a point of comparison. OK-total-IEV was the best blended satellite dataset, but the performance of the satellite SI datasets was close.

**Figure 5:**
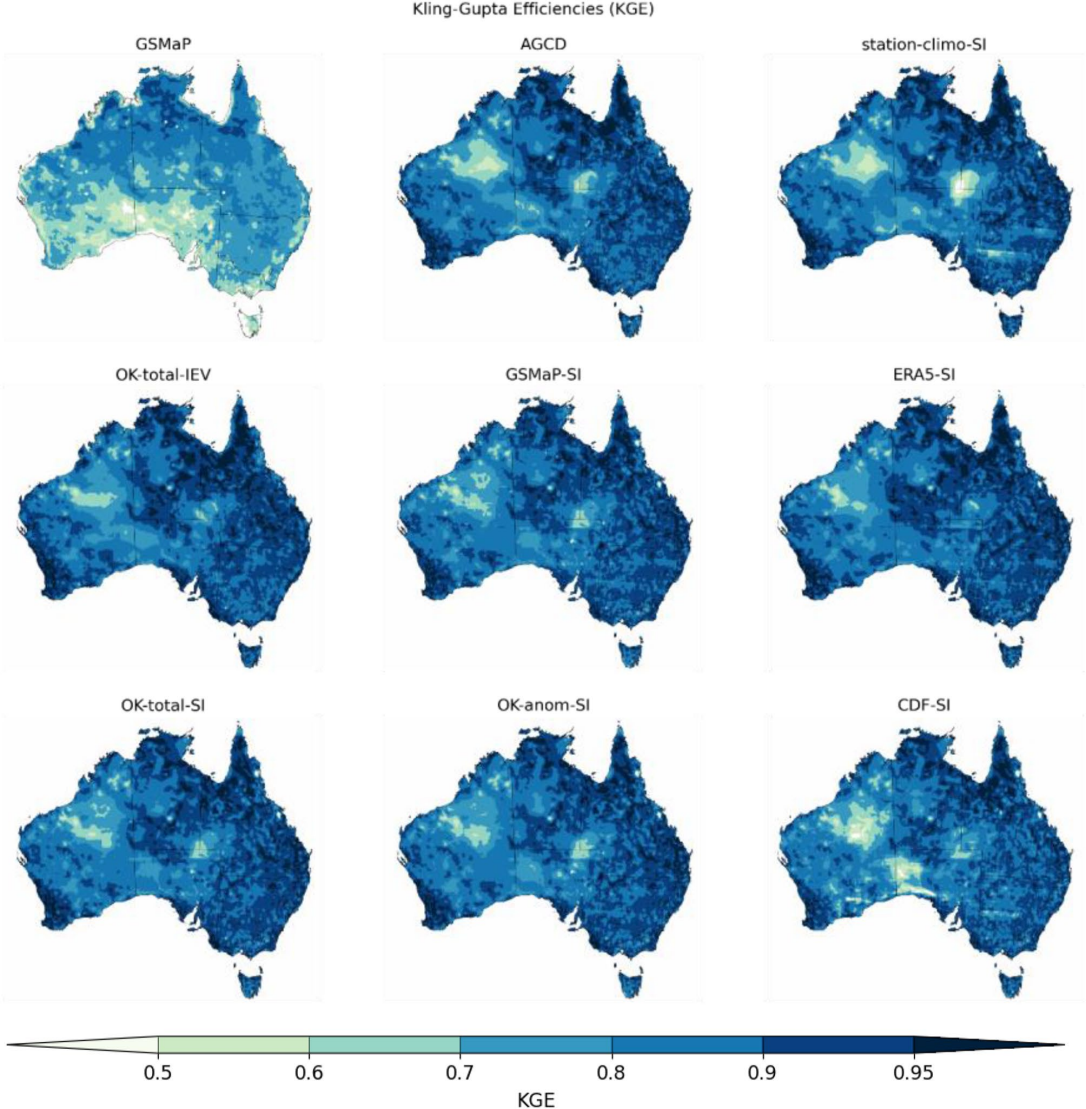
Kling-Gupta Efficiencies (KGE) of the datasets over the domain of Australia. A value of unity is ideal. The satellite-SI datasets have improved KGEs over northern interior of Western Australia, where gauge density is very low. Figure created using Matplotlib 3.3.3 on Python.

The KGE of each dataset is visualized spatially in Fig. 5. The improvement of the satellite SI datasets over GSMaP is evident except for the northern interior of Western Australia (WA) where the KGE falls to a minimum. This matches the in-situ validation where it was also indicated that this area possessed the highest error. The spatial distribution of KGE was similar amongst the operational AGCD (hereafter referred to as AGCD) and its low-resolution variant, apart from small regions over New South Wales. Both showed reductions in the KGE over the northern interior of WA and the junction of Northern Territory (NT), Queensland (QLD) and South Australia (SA). The use of satellite data led to a noticeable improvement in the KGE over these remote areas. An exception is CDF-SI which did not show much improvement over the northern interior of WA, in addition to a reduction in the KGE around the SA and WA border. Using ERA5 also produced a favorable result, particularly in increasing the KGE over the NT, QLD, and SA junction.

OK-total-IEV displayed a slightly better pattern of KGE than the satellite SI datasets. However, the overall similarity supports the idea that SI is producing a close to optimal result. A key difference was the presence of two very small regions of reduced KGE along the QLD coast that were present in the satellite SI datasets (and to a smaller degree, in AGCD), but which were not in OK-total-IEV. The SI process may be including two stations that have particularly different values to their surrounds, leading to information not represented in MSWEP.

**Figure 6:**
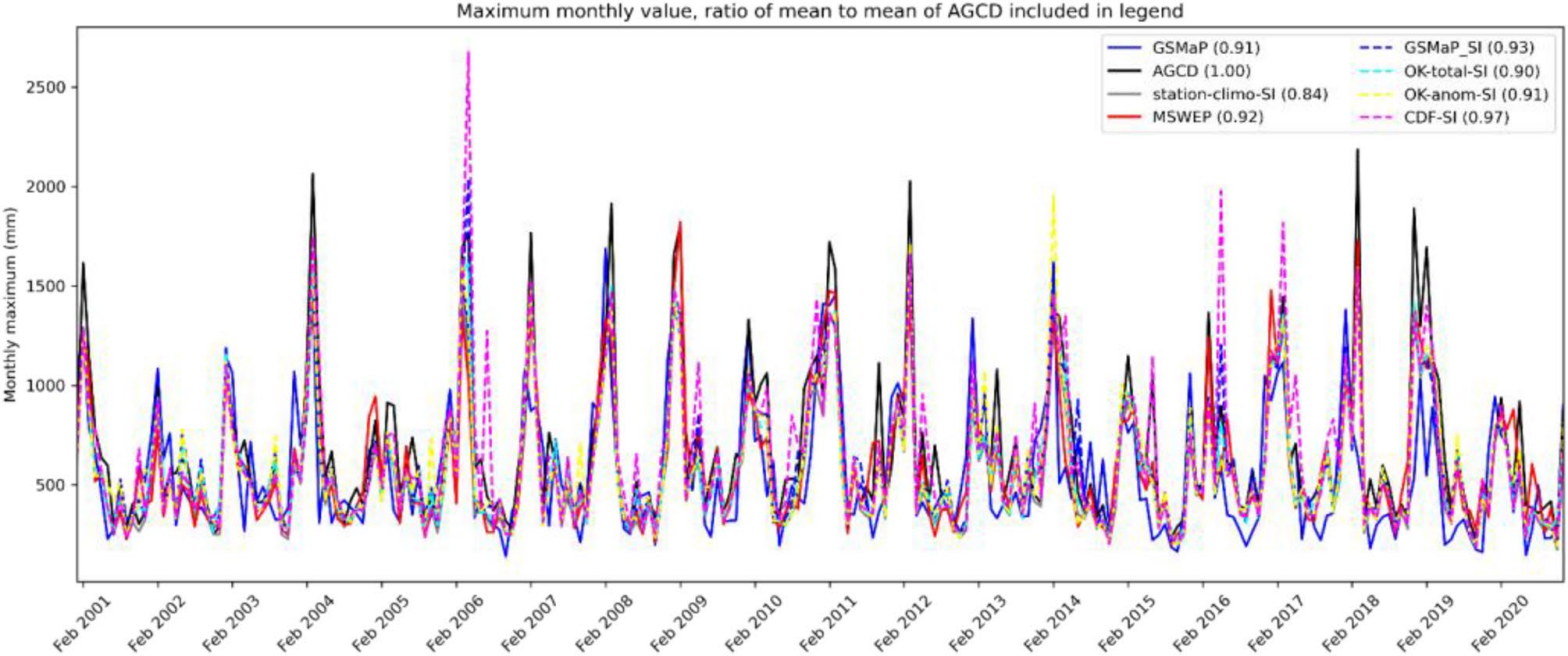
Time series of maximum monthly values from the satellite-SI datasets, AGCD and MSWEP. The ratio of the mean monthly maximum value to the mean monthly maximum value of AGCD is the number shown in the brackets of the legend.

Finally, given the identification of high-end rainfall is an important use of AGCD, a comparison of the time series of the maximum monthly values across the study period for the SI datasets against AGCD and MSWEP is shown in Fig. 6. A summary statistic in the form of the ratio of the mean maximum monthly value of the dataset against the mean maximum monthly value of AGCD is included in the legend.

The summary statistic shows that the mean maximum monthly value of AGCD is generally greater than that of the satellite-SI datasets and of MSWEP. It is evident from Fig. 6 that is in heavy rainfall months, where AGCD tends to noticeably have a greater value. It is possible that AGCD may be slightly overestimating the monthly maximum value. CDF-SI and OK-anom-SI have a few months where the maximum monthly value appears to be too large while GSMaP has months where the value appears to be too low.

#### A focus on a gauge-sparse region

**Figure 7:**
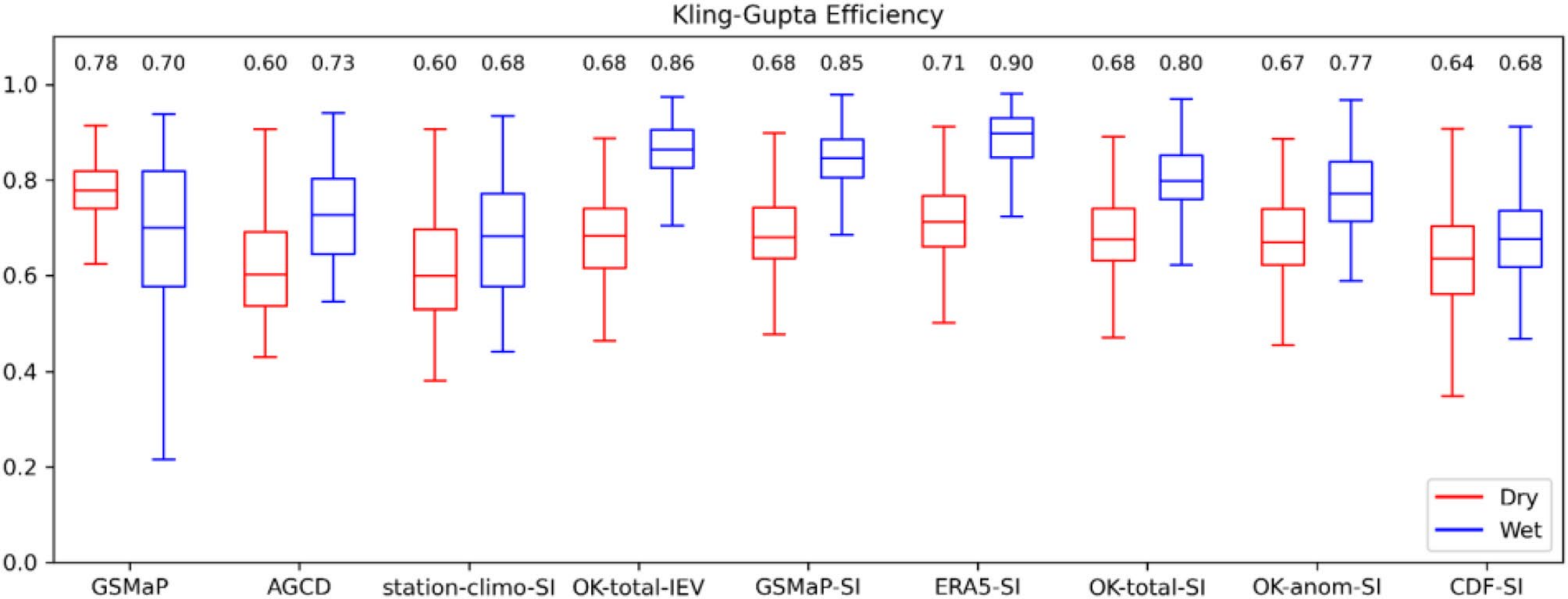
Boxplots of KGE across the datasets for a gauge-sparse sub-domain. The validation was split into the wet and dry seasons as well. A value of unity is ideal.

The general statistics were recalculated for a sub-domain to better compare the performance of the satellite-SI datasets over a gauge-sparse region. A sub-domain with latitude bounds between 20° S and 25° S, and longitude bounds between 121° E and 128° E was selected (see Fig. 1). This region has a gauge density of approximately 1 gauge per 200,000 km^2^ compared to the nationally averaged density of approximately 1 gauge per 1300 km^2^. As the region experiences a marked seasonal cycle in rainfall, the validation was also split into a wet season (October to April) and a dry season (May to September) components to examine if a seasonal difference in performance exists. The results are shown in Fig. 7.

Apart from CDF-SI, the satellite-SI datasets had higher KGEs than GSMaP, AGCD and station-climo-SI for both seasons. GSMaP-SI had the highest KGEs among the satellite-SI datasets, outperforming AGCD by + 8% in the dry season and by + 12% in the wet season. The improvement gained by using a satellite estimate as the first guess was much more noticeable over the gauge-sparse sub-domain than for the national domain, especially during the wet season. Presumably, this seasonal difference is partly because there is not as much rainfall during the dry season, and thus rainfall missed by using station climatology as a first guess is not as noticeable. The satellite-SI datasets also captured the variance in rainfall more accurately, with station-climatology-SI datasets exhibiting too little variance. This would be expected, as in the station-climatology-SI-datasets, this sub-region is heavily based on the interpolated climatology field. The individual components of the KGE for this analysis are included in Appendix B.

### Triple collocation analysis

**Figure 8:**
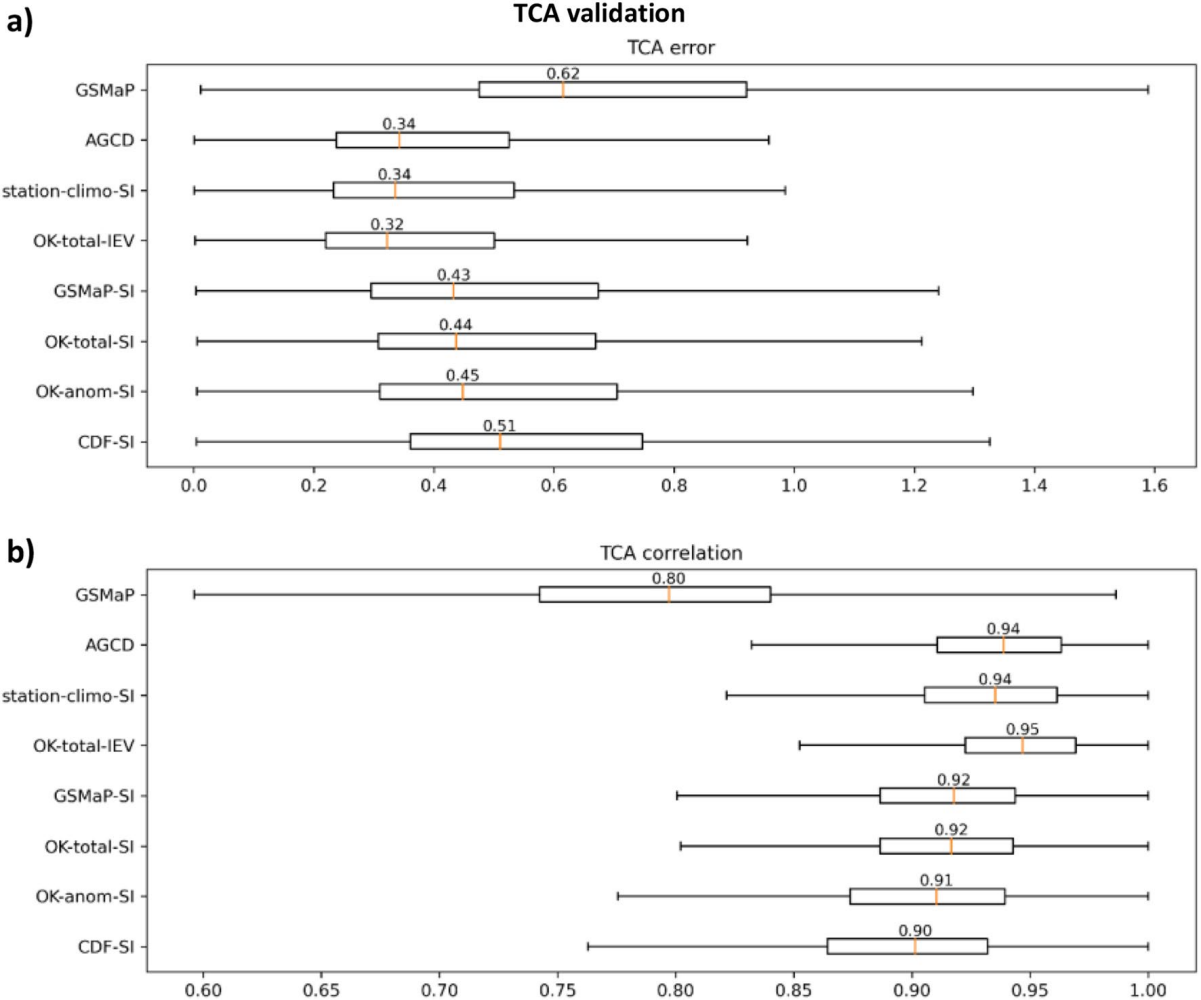
Boxplots of (**a**) correlation and (**b**) error statistics from TCA validation. The TCA error is in mm/day while TCA correlation is unitless. A value of 0 and unity is ideal for the error and correlation respectively. The boxes indicate the interquartile range (IQR), the whiskers extend out to the non-outlier minimum and maximums (Q1 − 1.5 × IQR and Q3 + 1.5 × IQR), and the line within the box represents the median.

The result of the triple collocation analysis is shown in Fig. 8.

The TCA validation suggests that amongst the SI datasets, station-climo-SI was the best-performing, followed by GSMaP-SI and OK-total-SI, then OK-anom-SI, and CDF-SI. It is clear the SI process improved GSMaP, though performance was slightly behind OK-total-IEV and the station climatology variants. The performance of station-climo-SI may have been slightly inflated by weaker satisfaction of the assumptions of error independence with ERA5, and of stationarity of errors (see Appendix C).

### Visual comparison

**Figure 9:**
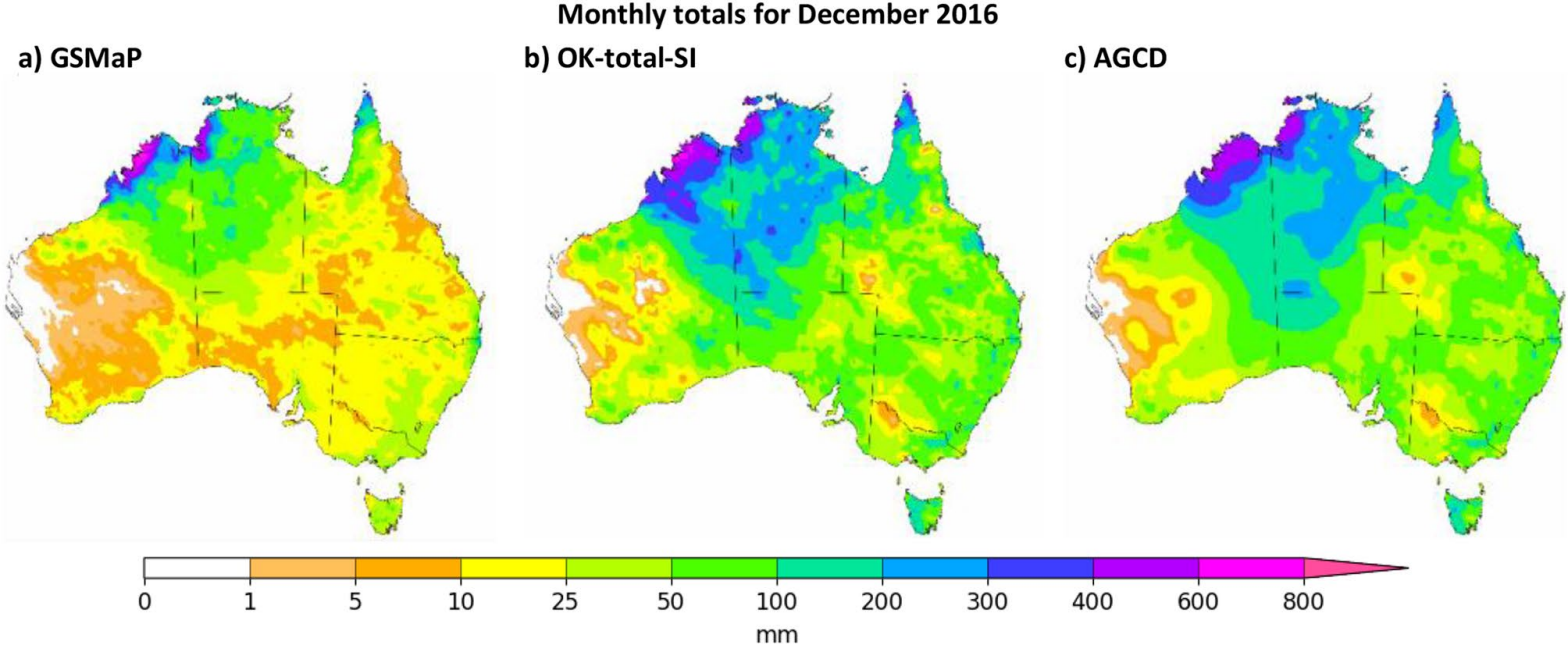
Visual comparison of December 2016 as represented by GSMaP-SI, OK-total-SI and AGCD. The greatest difference is over the north-east interior of WA and the south-west interior of NT where OK-total-SI, and to a lesser extent GSMaP-SI, have greater totals than AGCD. This is the region where ex-TC Yvette traversed after it crossed the coast, and is also where rain gauge density is extremely low. Figure created using Matplotlib 3.3.3 on Python.

**Figure 10:**
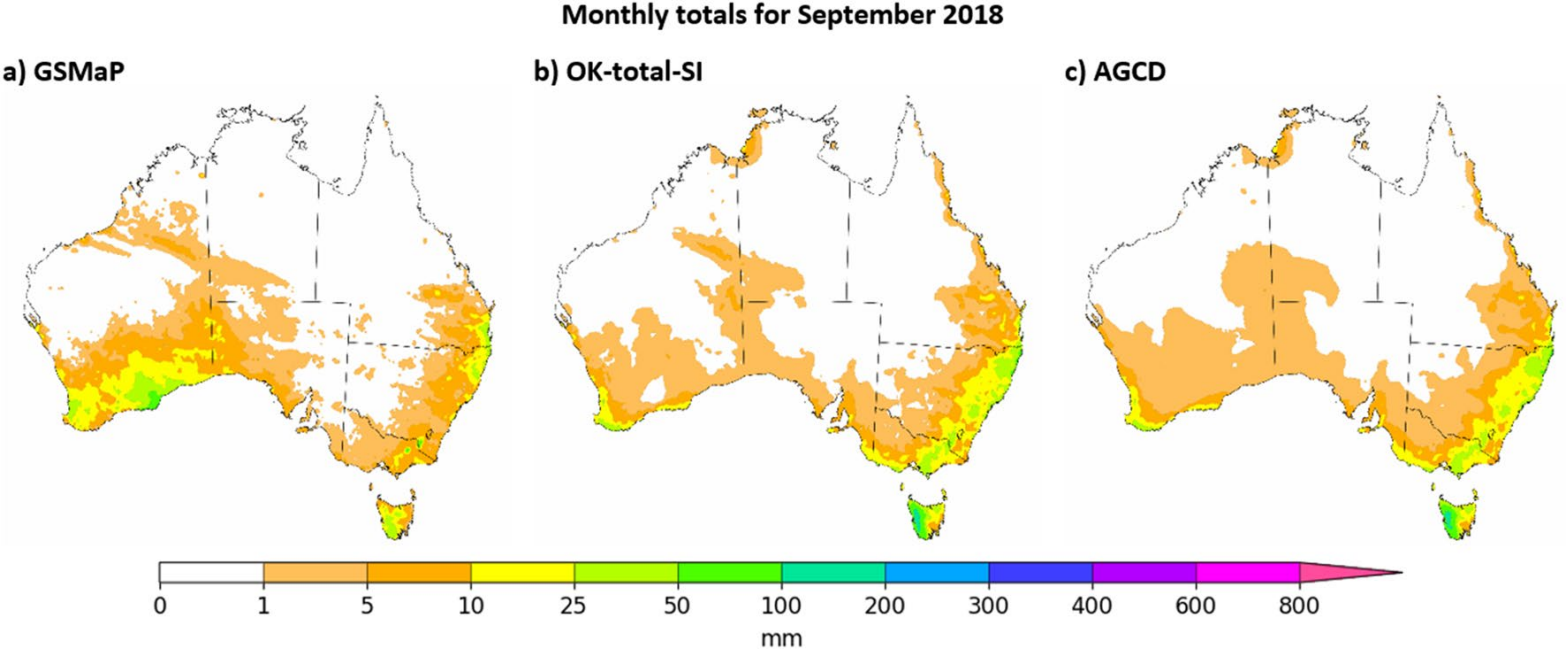
Visual comparison of September 2018 as represented by GSMaP-SI, OK-total-SI and AGCD. Figure created using Matplotlib 3.3.3 on Python.

To demonstrate the value of the satellite-SI datasets in an operational context, visualizations of OK-total-SI and GSMaP-SI are shown next to that of AGCD in Fig. 9 and Fig. 10. GSMaP-SI and OK-total-SI were chosen as they had the best overall performance from the three previous validations. The month of December 2016 is taken as an example of a month where SI was expected to produce significant upwards adjustment as it recorded significant rainfall over parts of the interior of Australia where gauge paucity was amongst the highest for the nation. The heavy rainfall over the interior was due to ex-tropical cyclone (ex-TC) Yvette crossing the coast near Broome on the 25th, and later tracking into central Australia where it combined with another tropical low to form a tropical inland depression^17^. At the time, it was the fifth-wettest December on record for Australia, the wettest for SA and the third-wettest for WA and NT^17^.

OK-total-SI was vastly different to GSMaP, with the former possessing higher amounts of rainfall than the latter over most of the domain except for the far northern coast of Western Australia. The rainfall in OK-total-SI was greater than AGCD over inland parts of WA and NT; these are regions where gauge density is extremely low (see Fig. 1). Given the low gauge density in these regions, AGCD would have relied heavily on station climatology, which was much lower than the rainfall experienced that month due to the anomalous inland tropical depression activity. In the face of two different representations, this gives reason to prefer the satellite-SI datasets. Elsewhere, the totals of OK-total-SI and AGCD were similar with only a few areas where modest differences are discernable, demonstrating SI has effectively adjusted the background field of GSMaP.

The area-averaged totals along with the monthly climatological ranking are reported regularly by the BOM as part of the national and state summaries. To demonstrate an example of how the use of satellite-SI datasets could improve operational products, the area-averaged totals for Australia, SA, WA and NT were re-calculated using OK-total-SI and compared to those from AGCD. There were some slight discrepancies between the AGCD averages and those presented officially by the BOM (hereafter referred to as AGCD-off). This was likely due to differences in processing techniques such as masking. To account for this, the percentage change between the averages of AGCD-off and AGCD for each region was used to scale OK-total-SI to form revised totals (hereafter referred to as OK-total-SI-off) that should be more consistent with AGCD-off. The values from OK-total-SI-off were then compared to the record of AGCD-off to obtain the climatological ranking of that month, with any change in the ranking being noted. The results are shown in Table 1.

**Table 1:** Area-averaged totals of AGCD and OK-total-SI for December 2016. OK-total-SI-off refers to an adjustment made to OK-total-SI to make it more equivalent to the official values presented by the BOM (AGCD-off).

	AGCD (mm)	OK-total-SI (mm)	Change (%)	AGCD-off (mm)	OK-total-SI-off (mm)	Rank change
Australia	91.57	94.76	+ 3.48	93.47	96.73	–
Western Australia	85.74	92.05	+ 7.36	87.72	94.18	+ 1
South Australia	66.90	62.65	− 6.35	68.50	64.15	–
Northern Territory	187.46	197.20	+ 5.20	188.46	198.25	–

The month of September 2018 is taken as an example of a month where SI was expected to produce significant downwards adjustment. According to AGCD data, this month was the driest month in the study period and the second-driest month (and driest September) since records began in 1900.

OK-total-SI was again noticeably different to GSMaP whilst being very similar to AGCD. The consistency between OK-total-SI and AGCD was greater than for December 2016, with the difference in their area-averaged totals being within 0.001% of each other. No change in the climatological ranking was achieved by using OK-total-SI over AGCD. AGCD had a smoother representation of rainfall though the general area where the monthly totals were greater than 1 mm was similar. Importantly, the patchiness in OK-total-SI could be more realistic given the very low amounts of rainfall involved.

The two examples demonstrate that the satellite-SI datasets can represent both upwards and downwards adjustment of rainfall in a similar manner to AGCD in gauge-dense areas.

## Discussion

### Difference between validations

The results consistently showed that the SI process was beneficial. However, there were discrepancies in the ranking of the SI datasets between the validation methods. An important question was whether the use of corrected or uncorrected GSMaP in the process was preferable. The in-situ and MSWEP validations suggested that the corrected GSMaP produced better performance while the TCA indicated otherwise. Performance between the datasets was marginal making changes in dataset rankings between the validation methods possible. Reference^18^ used similar triplets of ERA-Interim, Soil Moisture 2 Rain (SM2R) and satellite-based datasets and found matching rankings from TCA correlation with traditional Pearson correlations when the difference in performance between the datasets was marked. However, they also found that when the difference was small, the results were less conclusive. In Ref.^16^, it was identified that the use of corrected datasets in the blending process yielded better performance than using uncorrected datasets. This provides reason to question the ranking of GSMaP-SI over its corrected variants in the TCA.

The robustness of TCA is reliant on the accuracy of the datasets used in the triplets, and how well requisite assumptions are met. The use of radar^19^ or a gauge analysis^20^ would have been preferred to using SM2R given these remove the satellite dependency and are traditionally considered more accurate^15^. However, this was not possible, as radar coverage does not extend over all of Australia and the inclusion of gauge data in the blended satellite datasets exempted a gauge-based analysis in the triplet. Given both ERA5 and SM2R have some degree of reliance on satellite data (see Appendix C), this may be a reason why the TCA valued the use of uncorrected GSMaP in SI over the use of corrected versions.

Another difference was the best performing dataset, as well as the best performing SI dataset. In the in-situ and MSWEP validations, satellite SI datasets outperformed station-climo-SI, while in the TCA, the converse was true. The aforementioned discussion about the robustness of TCA is also valid for this point. We therefore believe greater credence should be given to the in-situ and MSWEP validations. This still leaves another discrepancy; the in-situ validation identified CDF-SI as a top performer, but it was worst-performing satellite-SI dataset in the other two validations.

This result introduces an important point in that our in-situ validation only considered bias statistics, whereas correlation and variability were also considered in our other validations. The KGEs for the satellite-SI datasets and the station-climo-SI dataset were similar. However, after analyzing the KGE components (see Appendix B), it was evident the satellite SI datasets had better bias and variability statistics than station-climo-SI, but worse correlation statistics. The apparent reduction in correlation could be influenced incorrectly by the use of MSWEP as truth. This is because MSWEP includes ERA5 reanalysis data, which is prone to spurious rainfall^21^. Consequently, rainfall areas in MSWEP are also likely to be overrepresented, skewing the correlation statistics. MSWEP is also known to be under-dispersed temporally^15^ and thus the finding that CDF-SI was the only dataset to record greater variability than MSWEP is notable. It is possible that CDF-SI may have actually represented variability more accurately than MSWEP but was penalized instead.

The fact that the datasets are largely similar also increases the likelihood of changes in rankings between the different validations. This is because the idiosyncrasies of each validation technique are more identifiable. For example, even though the in-situ validation was completed using a remove-one-station process, the majority of the stations are in coastal areas, therefore biasing this validation towards the coast. However, the MSWEP and TCA validations do not contain this bias. If the datasets were more disparate, the use of different validation techniques would be less likely to cause inconsistencies between rankings.

Finally, the apparent degradation in performance over the northern WA interior from the SI process will be addressed. A possible reason for this degradation is the positive bias introduced by the SI process over this region^22^. This was postulated to be due to the use of a minimum value of 2 mm for the background field. Given this region is capable of recording very low or even zero rainfall for a substantial part of the year, a slight positive bias can be quite detrimental to the correlation statistic as well. This is supported by the results from the gauge-sparse sub-domain analysis, where there were significant positive bias ratios in the SI datasets during the dry season (compared to only slight negative bias ratios in the wet season).

### Value of SI

Over the entirety of Australia, the best-performing dataset was somewhat inconclusive but the results demonstrated the SI process improved upon GSMaP, producing a dataset that matched or exceeded the performance of a version based on station climatology alone, as well as the operational AGCD dataset. When performance was considered over a gauge-sparse region, the improvement in using a satellite estimate as the background field was clearly noticeable, especially when there was significant rainfall such as over northern Australia during the wet season.

The MSWEP validation demonstrated the SI-datasets were slightly inferior to the blending technique we identified previously as best (OK-total-IEV). However, the SI method has some advantages over OK-total-IEV. Unlike OK-total-IEV, it does not need a gauge-analysis as an input. This benefit is compounded by the fact that using an uncorrected dataset still resulted in good performance, demonstrating that the absence of a gauge analysis for correction does not appear to be critical.

These points are beneficial in an operational context as they reduce computational resource and time requirements. Furthermore, in countries where a gauge analysis does not exist, the SI method should not be affected as much as other methods which rely on pre-blending correction to a gauge analysis. It is also more resistant to scenarios where a low-quality gauge analysis could cause issues. Given these benefits, along with only a slight reduction in performance compared to OK-total-IEV, the use of satellite data in SI should be considered a viable, if not valuable, blending technique.

## Conclusion

The AGCD rainfall dataset is the current operational rainfall analysis used in Australia. It provides a gridded representation of rainfall across the country that is useful for water availability assessment. However, being an interpolation of rain gauge data, its accuracy is limited over areas with low gauge density such as the nation's interior. The SI method used to generate AGCD is amendable to the inclusion of satellite data where it can be used in place of station climatology as the background field onto which monthly station observations are assimilated. This required modifying the algorithm to use only a single pass, converting the satellite data to a zero-mean centered anomaly field that could be ingested by SI, and tuning some of the parameters related to the interpolation process.

To determine the optimal input dataset for the process, uncorrected GSMaP dataset along with various corrected versions were used, in addition to the ERA5 reanalysis. The performance of the resultant products was compared against a low-resolution AGCD counterpart, as well as its higher resolution operational variant. The results demonstrated that the SI datasets made using satellite data and ERA5 had performance that was comparable or slightly better than the variants based purely on station data. However, in terms of performance over gauge-sparse regions, the satellite-SI and ERA5-SI datasets demonstrated a clear improvement with the KGE increasing by + 8% during the dry season and by + 12% during the wet season compared to AGCD. Considering biases that may have been present in the truth datasets used in the triple collocation analysis and gridded validations, the best satellite-based SI datasets appeared to be those produced from GSMaP corrected using quantile to quantile matching or through a linear correction to totals, though the use of uncorrected GSMaP also provided good results.

As operational analyses in Australia have only ever been purely generated from gauge data, this study's demonstration of the viability of using satellite data in SI, the current operational process, is an important step in advancing operational rainfall analyses in Australia.

## Methodology

### Data sources

The GSMaP dataset is generated by the Japan Aerospace Exploration Agency (JAXA) using rainfall estimates from Global Precipitation Mission (GPM) satellites. Passive microwave estimates are converted into rainfall rates using a forward model that links brightness temperatures to rainfall^23^. Cloud motion vectors formed using satellite infrared (IR) data are used to advect the microwave estimates spatiotemporally; in addition to the use of a Kalman Filter, this improves the accuracy of the dataset where microwave estimates are not available. The resultant dataset is known as GSMaP Moving Vector with Kalman Filter (GSMaP-MVK). This study uses GSMaP-Near Real Time-V6 (GSMaP-NRT-V6) as a base dataset. To form GSMaP-NRT-V6, GSMaP-MVK is calibrated to CPC Gauge Unified (a global gauge analysis) by roughly matching GSMaP-MVK to gauge estimates over land from the past 30 days, using a probability distribution function^24^.

GSMaP-NRT-V6 (hereafter referred to as GSMaP) was selected because it is one of the two best-performing satellite datasets currently available (and over some domains, it displays the best performance), with the other dataset being the Integrated Multi-SatellitE Retrievals for GPM (IMERG) datasets^14,25^. We elected to choose GSMaP over IMERG because GSMaP was easily accessible to us as part of the World Meteorological Organization's (WMO) Space-based Weather and Climate Extremes Monitoring (SWCEM) program. Furthermore, the modularity of this algorithm means the utilization of other satellite datasets (and more generally, other gridded datasets) as the background field could be achieved in the future, if desired.

GSMaP was further corrected to the Australian domain for this study using three methods:

- *Method 1: Linear correction to AGCD totals*. This method relied on finding the ratio of GSMaP data to AGCD at station locations. These ratios were then converted to a grid using Ordinary Kriging (OK) which was applied back onto the GSMaP data to correct it.
- *Method 2: Linear correction to AGCD anomalies*. Instead of finding the ratio between the totals, the ratios of the anomalies of GSMaP and AGCD (with respect to their own climatology) were found. The resultant correction grid was used to correct the GSMaP anomalies which were added back onto the climatology to form the totals.
- *Method 3: Quantile to quantile correction to AGCD*. At each grid point, a cumulative distribution function (CDF) was formed from the monthly climatological record of both GSMaP and AGCD. A Gamma distribution was then fitted to both CDFs and quantile-to-quantile matching was performed to adjust the GSMaP value so that its frequency corresponded to that in the AGCD record.

Further detail on these three methods can be found in Ref.^16^ where a variety of correction and blending techniques were evaluated over Australia. A blended dataset using GSMaP linearly corrected to AGCD totals that was subsequently blended with AGCD via inverse error variance weighting, performed the best (OK-total-IEV). However, the ranking of the best corrected dataset varied between the blending techniques and so it was prudent to trial all three correction methods again in this study. These corrected GSMaP datasets (referred respectively to as OK-total, OK-anom and CDF) were used in the SI algorithm along with uncorrected GSMaP. The resultant datasets are labelled OK-total-SI, OK-anom-SI, CDF-SI and GSMaP-SI.

To use satellite-based datasets as the background field in the SI process, some pre-processing had to be performed. Firstly, the monthly satellite precipitation estimates were bilinearly interpolated from 0.25° to 0.1° to allow a dataset with 0.1° resolution to be created after SI. Extra detail was not derived from this finer background field, but rather from the SI process where station information at a higher resolution could now be used due to upscaling of the background field.

Secondly, the monthly field had to then be converted to a zero-mean-centered anomaly field. An anomaly field was created by dividing the monthly estimate by its climatological mean. This climatology was computed from the 20 years of satellite data from 2001 to 2020. The WMO recommends that the climatological record should have contained at least 30 years to better account for climatological extremes but the GSMaP satellite record is limited to post-2001. Unity was then subtracted from this anomaly field to center the anomaly field to zero, i.e., a value of zero indicates a monthly value that equals climatology. This zero-mean-centered anomaly field was then used as the background field.

### SI algorithm

The process of SI used to create the operational AGCD was outlined in “Introduction”. This section provides some additional details relevant to the study though readers are referred to Ref.^13^ for a more thorough account. In the operational method, two SI passes are performed in order to handle the irregularity that would otherwise be introduced in gauge-sparse areas. The first pass creates a low-resolution 0.25° × 0.25° analysis from which the gauge network density can be determined. A second pass is then performed on this coarse analysis to create a final high-resolution 0.01° × 0.01° analysis that uses all available observations. In regions where data paucity is determined to be extreme, pseudo-observations are introduced so the result from the second pass is a smooth analysis.

For this study, the process was reduced to a single pass to acknowledge how the 'first guess' of corrected satellite data was already an adequate estimate of rainfall and did not require pseudo-observations. The resolution of the final product was also decreased from 0.01° × 0.01° to 0.1° × 0.1°, reflecting the native resolution of GSMaP.

Furthermore, some parameters in the process were changed. These parameters were:

- *Typical length scale* The SI process uses station covariance information based on a variogram that is computed from all available stations. This parameter refers to the proportion of the variogram that is used. A value of 0.3 was used indicating only the closest 30% of the stations could be considered. This sets the dimensions of the correlation matrix.
- *Sector length fraction* The analysis is comprised from many smaller sectors where the number of stations in each sector must not exceed the limit set by the dimensions of the correlation matrix. This parameter refers to the fraction of this number that must be in the central sector. A value of 0.25 was used meaning 25% of the stations must have been within the central sector with the rest being able to be drawn from surrounding sectors.
- *Sector length extension* Each sector is allowed to overlap other sectors to ensure smoothness in the analysis. This parameter refers to the maximum amount of overlap that can occur. A value of 0.3 was used meaning 30% of each sector could be extended.
- *Max side* This refers to the maximum number of grid points that a sector can use. A value of 48 was used.

### Validation

Validation of the monthly datasets was performed on a monthly scale over the study period of 2001 to 2020. The optimal interpolation algorithm provided cross-validation statistics. During the generation of the analysis, individual stations were removed with the analysis being recomputed to minimize the error variance to the removed observation. The error variance of the removed observation was then checked against a tolerance to determine if that observation was considered erroneous. These statistics were used to compare the various SI-generated datasets in a form of 'remove one station' in-situ validation.

Additionally, the Kling-Gupta Efficiency (KGE) value was calculated as part of a gridded validation using the Multi-Source Weighted-Ensemble Precipitation (MSWEP) dataset as truth. MSWEP is a blended dataset formed from ERA5 model reanalysis, the Integrated Multi-satellite Retrievals for GPM (IMERG) satellite estimates and station values^26^. The KGE facilitates an uncomplicated analysis of the correlation, bias, and variability of a dataset. Reference^27^ introduced it as an easy-to-interpret metric that decomposes key components of performance with^28^ modifying it slightly to remove cross-correlation between the bias and variability components. The KGE is dimensionless and defined by the equations:

1 $$KGE=1-\sqrt{{\left(r-1\right)}^{2}+{\left(\beta -1\right)}^{2}+{\left(\gamma -1\right)}^{2}},$$

2 $$\beta =\frac{{\mu }_{i}}{{u}_{t}},$$

3 $$\gamma =\frac{C{V}_{i}}{C{V}_{t}}=\frac{{\sigma }_{i}/{\mu }_{i}}{{\sigma }_{t}/{\mu }_{t}},$$

where r is the Pearson's correlation between dataset being verified and the dataset taken as truth, β is the bias ratio, γ is the variability ratio, μ is the mean precipitation, and the subscripts i and t represent the dataset being verified and the truth dataset respectively.

The components are computed at each grid point in the domain with the median being calculated across the domain.

Finally, a triple collocation analysis (TCA) to two non-gauge-based datasets was performed to capture performance away from stations. ERA5 and Soil Moisture 2 Rain (SM2R) data in addition to the dataset being verified were used to form the data triplet. The use of ERA5 in the triplet exempted ERA5-SI from this validation. ERA5 is the fifth-generation climate model reanalysis from the European Centre for Medium Range Weather Forecasts (ECMWF) and is generally considered the best-performing reanalysis^15,29^. SM2R is a gridded rainfall dataset back-calculated from satellite-derived soil moisture estimates with performance similar to ERA5^30^. These two datasets are chosen for the TCA due to their good performance and relatively high independence from the datasets being verified.

TCA enables a comparison of the relative ranking of three independent datasets in the absence of a known 'truth'. It was introduced for error estimation of wind data by Ref.^31^ and has since seen usage in the verification of other fields where the 'truth' can be difficult to ascertain, including rainfall^18,32^. Reference^16^ found it yielded results consistent with traditional validation over Australia. The relevant equations and assumptions will be outlined below; for a detailed overview see Ref.^33^.

The technique yields error variances (σ), as well as correlations (ρ) as defined by:

4 $$\sigma =\begin{array}{c}\sqrt{{Q}_{11}-\frac{{Q}_{12}{Q}_{13}}{{Q}_{23}}}\\ \sqrt{{Q}_{22}-\frac{{Q}_{12}{Q}_{23}}{{Q}_{13}}}\\ \sqrt{{Q}_{33}-\frac{{Q}_{13}{Q}_{23}}{{Q}_{12}}}\end{array},$$

5 $${\rho }_{t,X}=\begin{array}{c}\sqrt{\frac{{Q}_{12}{Q}_{13}}{{Q}_{11}{Q}_{23}}}\\ sign({Q}_{13}{Q}_{23})\sqrt{\frac{{Q}_{12}{Q}_{23}}{{Q}_{13}{Q}_{22}}}\\ sign({Q}_{12}{Q}_{23})\sqrt{\frac{{Q}_{13}{Q}_{23}}{{Q}_{12}{Q}_{33}}}\end{array},$$

where Q represents the covariance between a pair of datasets and the subscripts 1,2,3 are used to distinguish between individual datasets with t being the unknown 'truth'.

There is a sign ambiguity in Eq. (5) but the datasets can be safely assumed to be positively correlated with the 'truth'^18^. The assumptions underlying the principle of TCA are: (1) linearity between the truth and the datasets, (2) stationarity of the truth and its errors and (3) independency amongst the errors and between the error and truth. The climatology-removed time series are used to reduce non-linearity between the datasets due to different climatologies^33^. Appendix C presents the degree to which these assumptions are met and thereby the suitability of TCA in this study though it should be noted that traditional validation metrics such as the RMSE and Pearson's correlation can also be afflicted by violations in these assumptions^33^.

Several other datasets were included as references. The SI algorithm used in this study was also trialed with background fields derived from:

- *Station climatology* The generated dataset is very similar to the operational AGCD dataset but with slight differences due to being at a lower resolution and from the changes to the algorithm mentioned in “SI algorithm”.
- *ERA5* This was performed to investigate the differences in using a reanalysis instead of satellite data.

**Figure 11:**
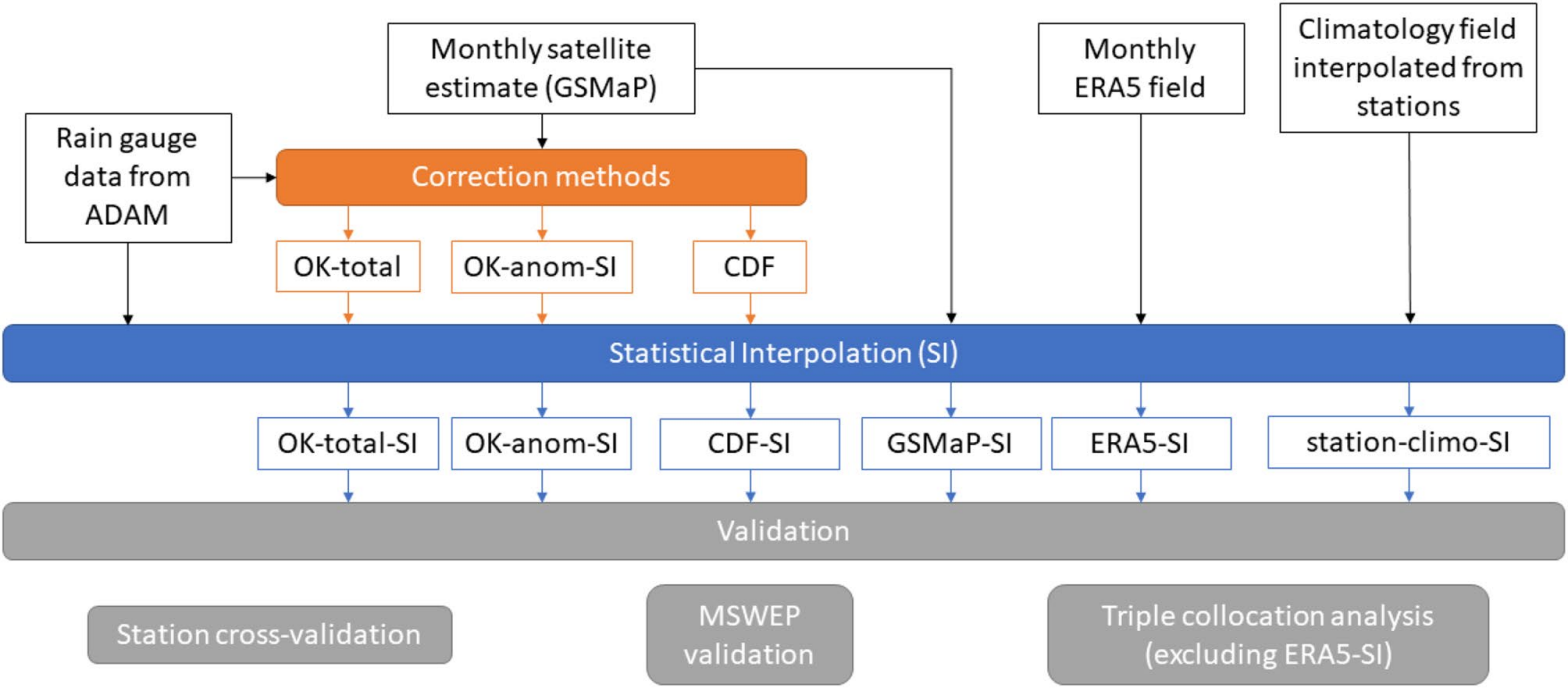
Schematic of the workflow. Input datasets are shown in the black boxes, corrected satellite datasets are shown in orange boxes, datasets produced by statistical interpolation (SI) are shown in blue boxes.

These datasets are referred to as station-climo-SI and ERA5-SI respectively. Furthermore operational AGCD and total-OK-IEV, the most performant blended dataset from Ref.^16^ were included as references for the TCA and MSWEP validations. To ensure greater independence from MSWEP, the version of total-OK-IEV that uses ERA5 to calculate the error variance-based weights was used in the MSWEP validation. Likewise in the TCA, the MSWEP-based version was used. Reference^16^ demonstrated the difference between the two versions is slight and did not affect overall rankings. These datasets are not present in the in-situ validation as the results from that validation are an output from the SI process. A schematic of our workflow is shown in Fig. 11.

For a summary of the datasets used in this study, readers are referred to Table A2 in Appendix D.

## Supplementary Information

Supplementary Information.

## Data Availability

GSMaP data were provided by EORC, JAXA and can be obtained by registering as a user at https://sharaku.eorc.jaxa.jp/GSMaP/index.htm. AGCD and station gauge data were provided by the Bureau of Meteorology and can be obtained from http://dx.doi.org/10.25914/6009600786063. Contains modified Copernicus Climate Change Service Information (2019) due to use of ERA5 data which can be obtained from https://cds.climate.copernicus.eu/cdsapp#!/dataset/reanalysis-era5-single-levels-monthly-means. Neither the European Commission nor ECMWF is responsible for any use that may be made of the Copernicus Information or Data it contains. MSWEP data were provided by GloH2O which can be obtained by seeking permission from http://www.gloh2o.org/mswep/ (2019).
