# Author Correction: Enhancing the Australian Gridded Climate Dataset rainfall analysis using satellite data

**DOI:** 10.1038/s41598-023-28997-z

**Published:** 2023-01-31

**Authors:** Zhi‑Weng Chua, Alex Evans, Yuriy Kuleshov, Andrew Watkins, Suelynn Choy, Chayn Sun

**Affiliations:** 1 Bureau of Meteorology, Melbourne, VIC Australia; 2 Royal Melbourne Institute of Technology, Melbourne, VIC Australia

Correction to: *Scientific Reports* 10.1038/s41598-022-25255-6, published online 30 November 2022

The original version of this Article contained an error in Figure 10, where the caption of the figure was incorrectly given as ‘Monthly totals for December 2016’. The correct caption now reads: ‘Monthly totals for September 2018’.

The original Figure 10 and accompanying legend appear below.

**Figure 10:**
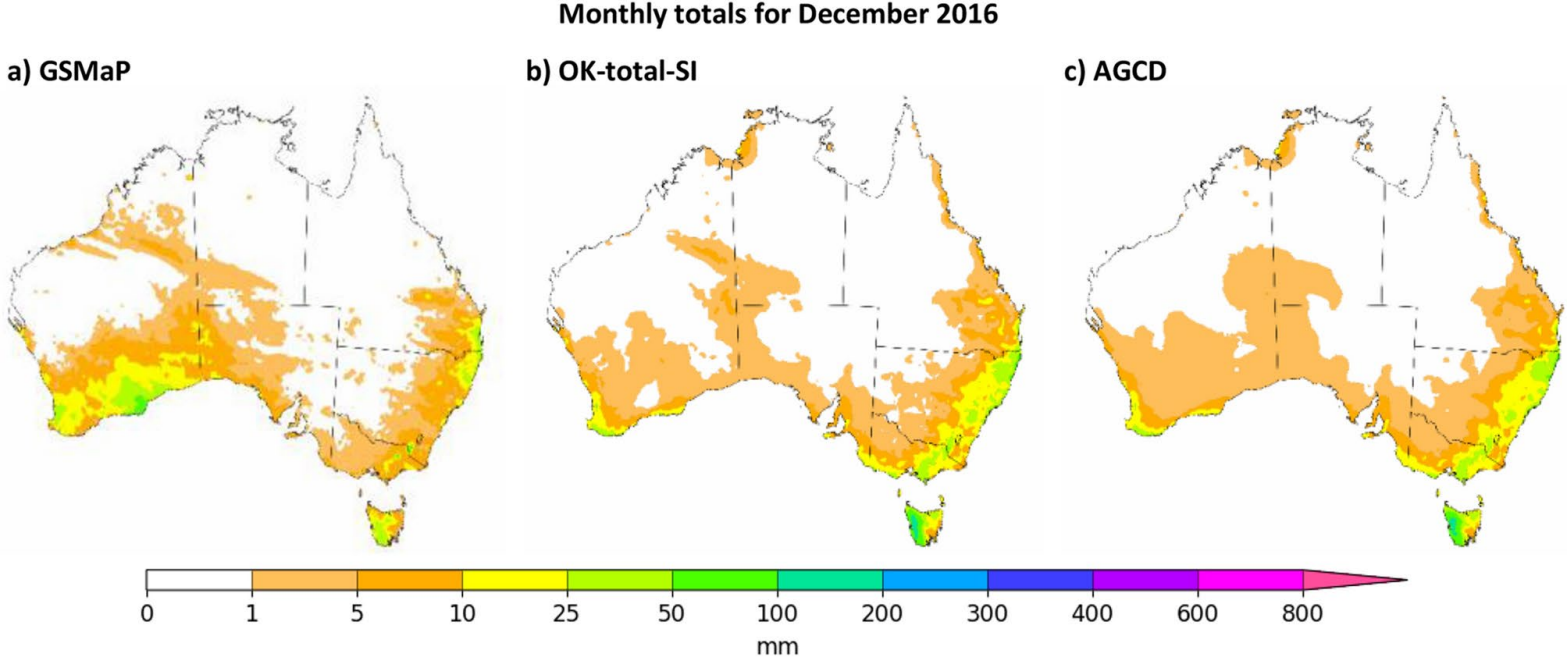
Visual comparison of September 2018 as represented by GSMaP-SI, OK-total-SI and AGCD. Figure created using Matplotlib 3.3.3 on Python.

Additionally, in the Results section, under the subheading ‘Visual comparison’,

“To demonstrate the value of the satellite-SI datasets in an operational context, visualizations of OK-total-SI and GSMaP-SI are shown next to that of AGCD in Fig. 9.”

now reads:

“To demonstrate the value of the satellite-SI datasets in an operational context, visualizations of OK-total-SI and GSMaP-SI are shown next to that of AGCD in Fig. 9 and Fig 10.”

Finally, in the Methodology section, under the subheading ‘Validation’,

“A schematic of our workflow is shown in Figs. 10 and 11.”

now reads:

“A schematic of our workflow is shown in Fig. 11.”

The original Article has been corrected.

