# Supplementary material for: Enhancing the Australian Gridded Climate Dataset rainfall analysis using satellite data
Source: Sci Rep. 2022 Nov 30;12:20691. doi: 10.1038/s41598-022-25255-6 (PMC9712511; doi:10.1038/s41598-022-25255-6)
Supplement: Supplementary file 1 — Supplementary Information. [file 41598_2022_25255_MOESM1_ESM.docx]

Supplementary material - Appendices

Appendix A

Time series of the months which form the boxplots of Figure 2 are presented below in Figure A1.

| **Time series of mean bias, mean absolute error and root-mean-squared-error for the in-situ validation over the entire Australian domain** |
| --- |
| **a)**  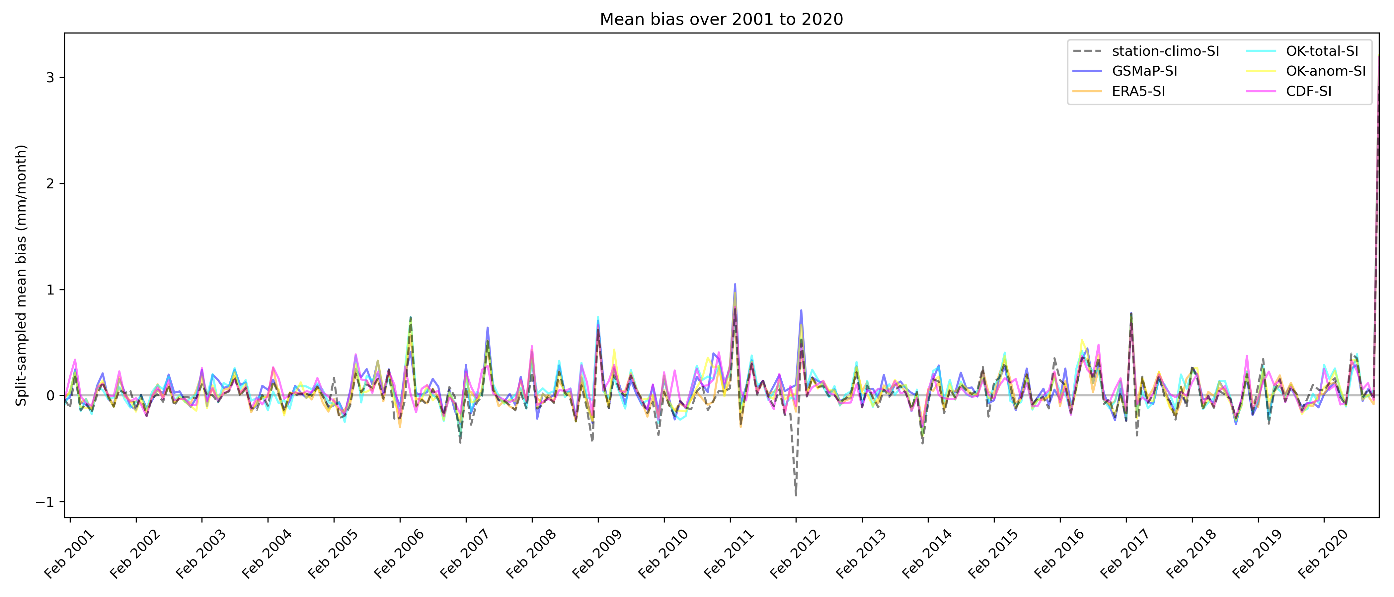 |
| **b)**  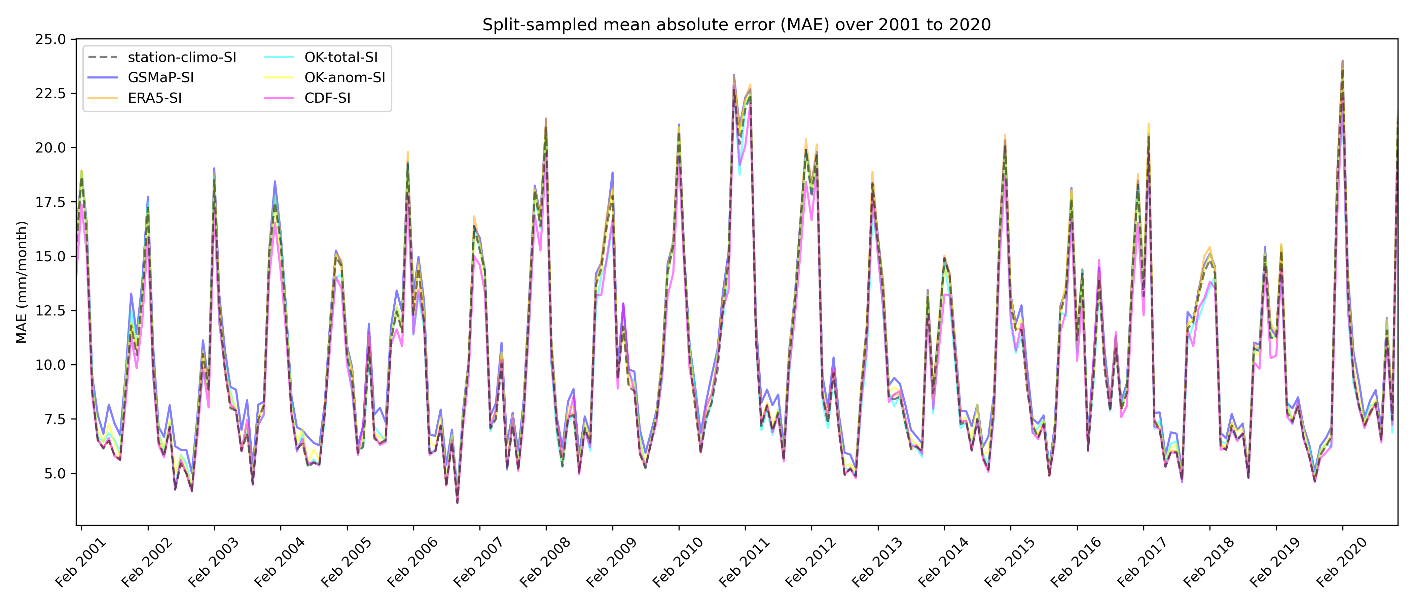 |
| **c)**  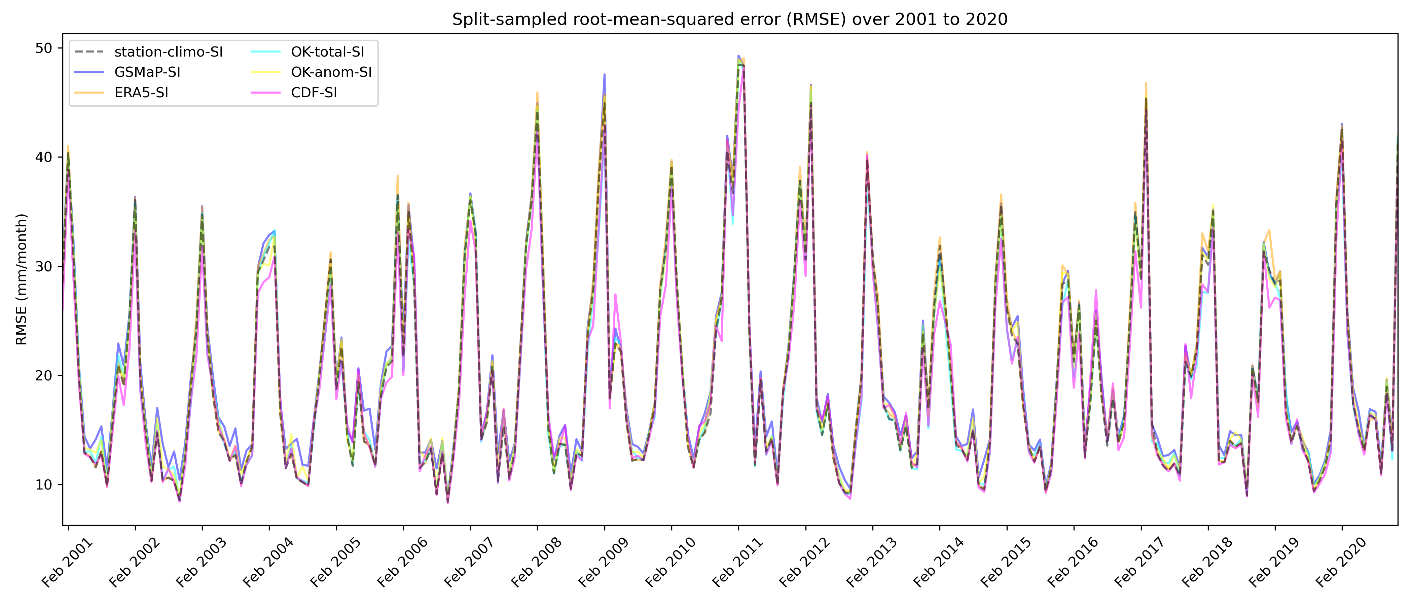 |

**Figure A1.** Time series of the a) mean bias, b) mean absolute error and c) root-mean-squared-error from the split-sample in-situ validation over the entire Australian domain. A value of zero is ideal for all three metrics.

The satellite-SI datasets tend to track AGCD closely for all three metrics. CDF-SI has the greatest disparity amongst the datasets, having a greater error than AGCD for most months though there are months where it has a smaller error.

There is a clear seasonality to the MAE and the RMSE with these values becoming significantly greater during the Austral summer. However, there is little correlation between the error magnitude (RMSE and MAE) and the error sign (MB).

Appendix B

Boxplots of the components of the KGE from validation over the entire Australian domain (see Figure 4) are presented below in Figure A2.

| **KGE components over entire Australian Domain** |
| --- |
| **a)**  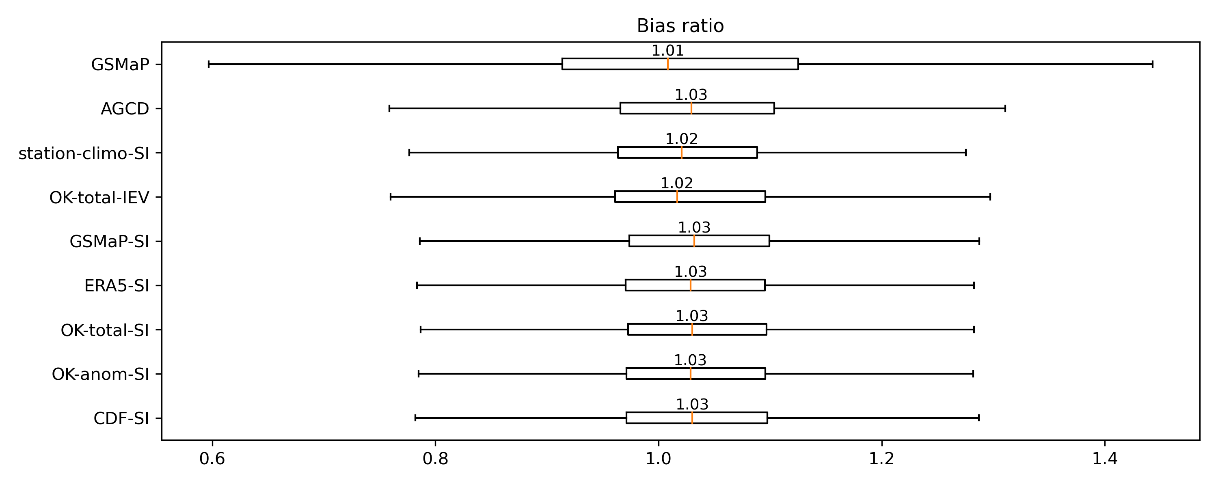 |
| **b)**  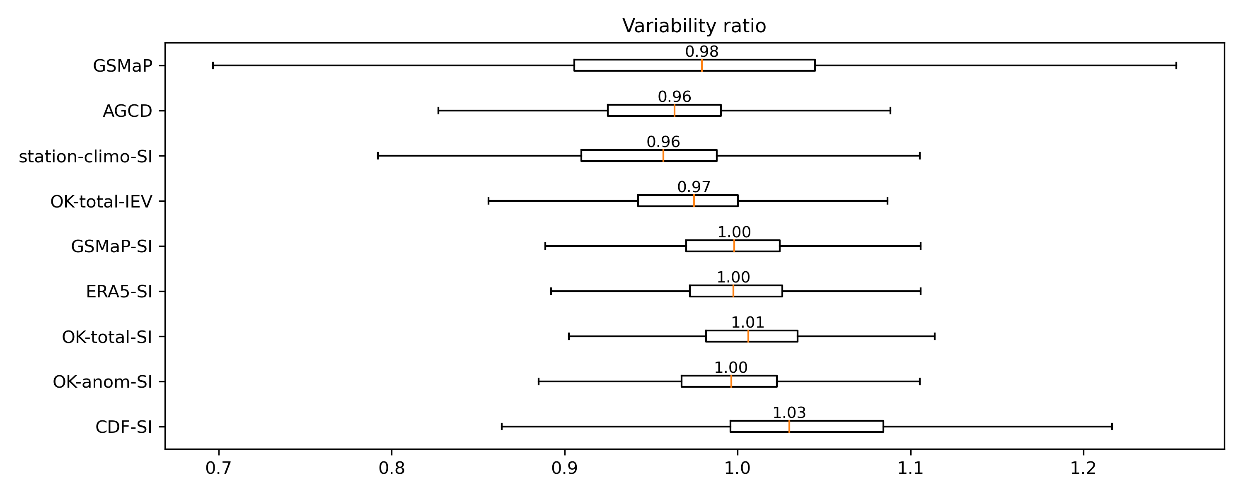 |
| **c)**  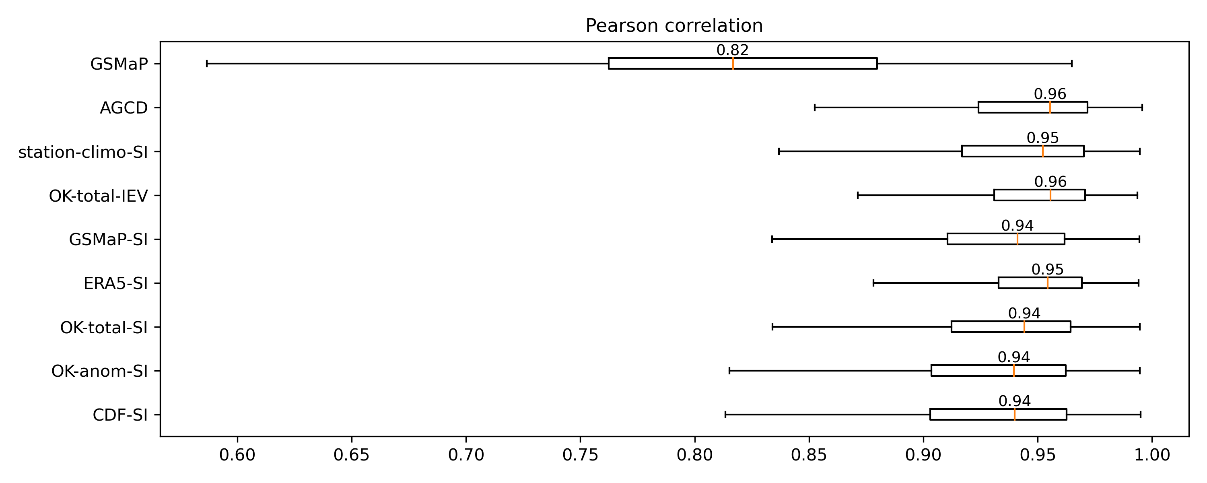 |

**Figure A2.** Boxplots of the a) bias ratio, b) variability ratio and c) Pearson correlation from the MSWEP validation over the entire Australian domain. A value of unity is ideal for all three metrics. The boxes indicate the interquartile range (IQR), the whiskers extend out to the non-outlier minimum and maximums (Q1 - 1.5 × IQR and Q3 + 1.5 × IQR), and the line within the box represents the median.

Box plots of the components of the KGE from validation over the gauge-sparse sub-domain (see Figure 6) are presented below in Figure A3.

| **KGE components over entire a gauge-sparse sub-domain** |
| --- |
| **a)**  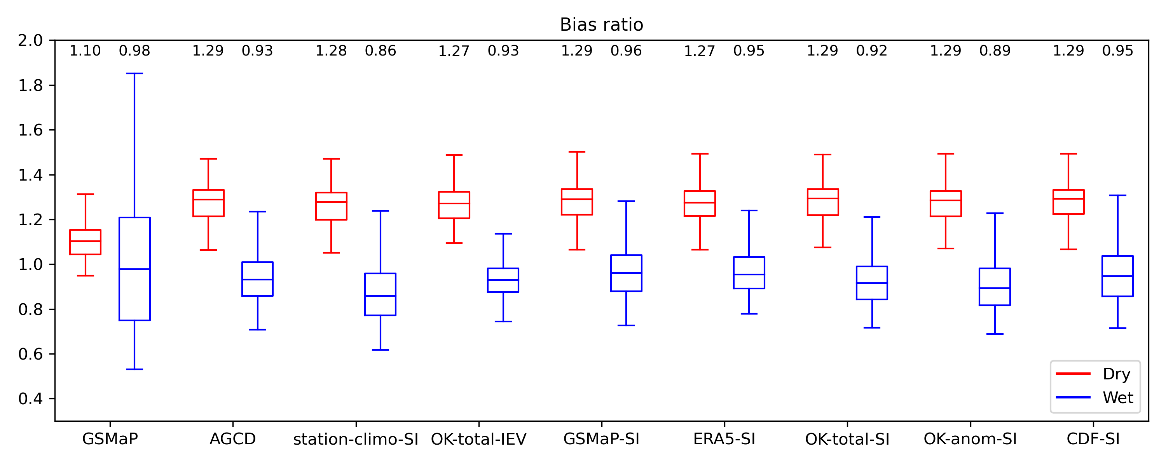 |
| **b)**  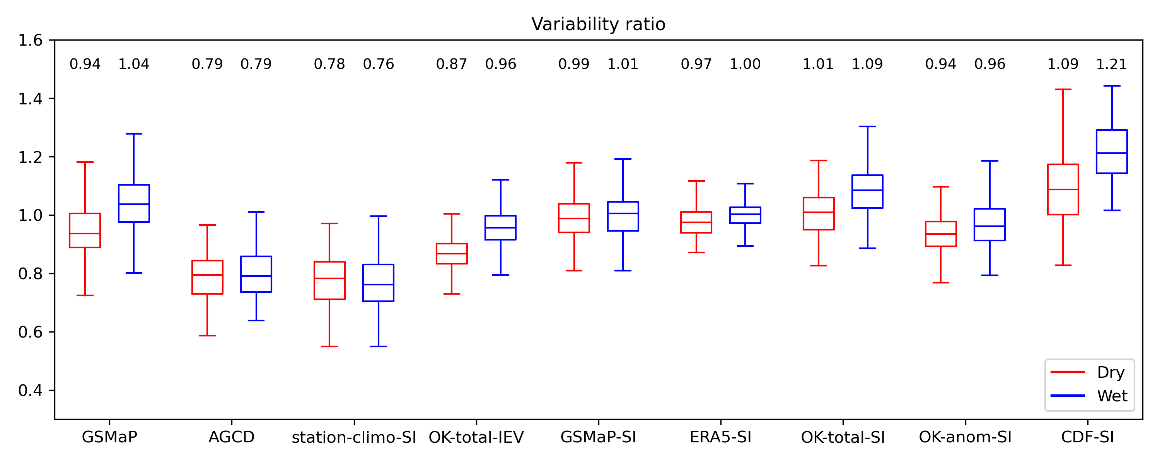 |
| **c)**  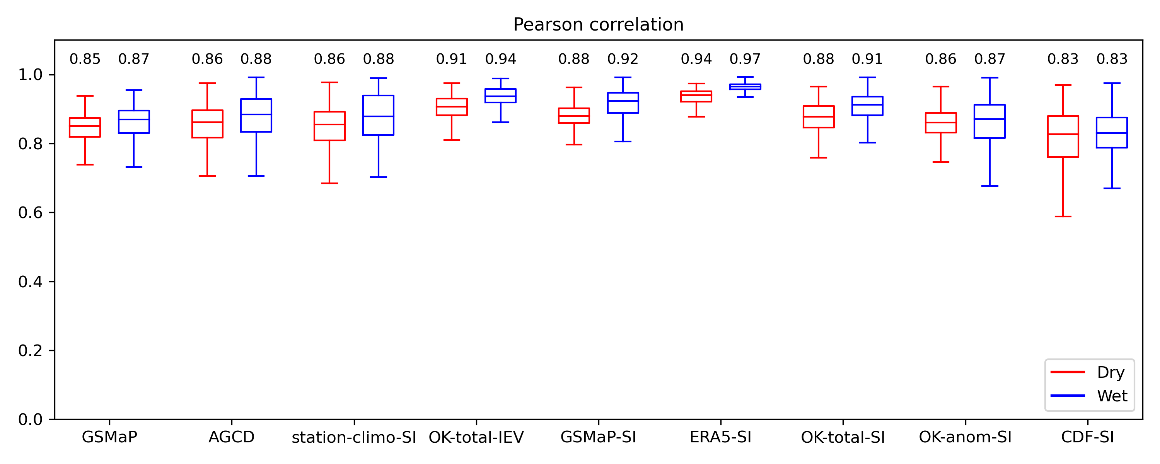 |

**Figure A3.** Boxplots of the a) bias ratio, b) variability ratio and c) Pearson correlation from the MSWEP validation over the gauge-sparse sub-domain. A value of unity is ideal for all three metrics. The boxes indicate the interquartile range (IQR), the whiskers extend out to the non-outlier minimum and maximums (Q1 - 1.5 × IQR and Q3 + 1.5 × IQR), and the line within the box represents the median.

Appendix C

The degree to which the datasets used in the TCA satisfy the assumptions requisite for TCA are demonstrated here.

1. Orthogonality of errors (the expected sum of the errors is zero).

A time-series of errors for each dataset was computed by using MSWEP as truth and subtracting MSWEP from the dataset. The error time series was then compared to the mean time series to evaluate its relative magnitude. The bias is generally small. Most of the datasets have a mean bias that is around 3% or less of the mean signal. SM2R and ERA5 have slightly bigger biases (around 7% and 5% of the mean signal).

1. There is no cross-correlation amongst the error of the datasets, as well as with the truth.

A linear correlation of the error time series of each dataset was computed against the error time series of ERA5 and SM2R. Correlations between errors are low, with the highest value being around 0.4. Perfect independence is unrealistic due to the existence of common factors that affect accuracy between the datasets (e.g., topography). SM2R contains gauge influence from its bias correction to gauges while ERA5 ingests moisture-related information (but not precipitation estimates) from satellites.

1. Stationarity of data.

An Augmented Dickey-Fuller Test (ADFT) was performed on both the monthly time series and the error time series of each of the datasets used in TCA. The ADFT tests the null hypothesis that a unit root exists in the dataset, thereby indicating non-stationarity ^33^. More negative test values indicate greater confidence the dataset is stationary. Generally, the datasets demonstrate a high degree of stationarity.

1. The datasets can be linearly related to each other.

The linear correlation between the time series of each dataset was computed. All the datasets demonstrate a very high linear correlation to each other.

**Table A1.** Metrics testing whether the assumptions required for TCA are satisfied.

| **Dataset** | **ADF Statistic** | | ***p*-Value** | | **Ratio of Bias to Mean (%)** | **R to SM2R** | | **R to ERA5** | |
| --- | --- | --- | --- | --- | --- | --- | --- | --- | --- |
|  | Raw | Bias | Raw | Bias |  | Raw | Bias | Raw | Bias |
| SM2R | −2.76 | -11.08 | 0.06 | 0.00 | −7.25 | - | - | 0.96 | 0.18 |
| ERA5 | −2.49 | -7.92 | 0.12 | 0.00 | −5.14 | 0.96 | 0.18 | - | - |
| AGCD | −2.39 | -2.69 | 0.15 | 0.08 | 2.24 | 0.97 | 0.19 | 0.97 | -0.36 |
| GSMaP | −2.14 | -11.86 | 0.23 | 0.00 | −0.69 | 0.96 | 0.24 | 0.99 | 0.36 |
| OK-total-IEV | −2.41 | -2.35 | 0.14 | 0.16 | 1.62 | 0.97 | 0.17 | 0.99 | 0.36 |
| GSMaP-SI | -2.37 | -2.51 | 0.15 | 0.11 | 1.96 | 0.97 | 0.26 | 0.99 | 0.36 |
| station-climo-SI | -2.34 | -2.15 | 0.16 | 0.22 | 3.09 | 0.97 | 0.19 | 0.99 | 0.40 |
| OK-total-SI | -2.42 | -2.37 | 0.13 | 0.15 | 2.67 | 0.97 | 0.18 | 0.99 | 0.32 |
| OK-anom-SI | -2.35 | -3.07 | 0.16 | 0.03 | 2.55 | 0.98 | 0.26 | 0.99 | 0.40 |
| CDF-SI | -2.43 | -2.21 | 0.13 | 0.20 | 2.97 | 0.97 | -0.00 | 0.99 | 0.30 |

Overall, the datasets satisfy the assumptions required. The degree of satisfaction is weakest for station-climo-SI where the assumptions of error independence to ERA5 and error stationarity are weaker.

**Appendix D**

**Table A2.** Summary of datasets used in this study.

| Dataset, provider | Resolution | Use in this study | Dataset description |
| --- | --- | --- | --- |
| AGCD, BOM | 0.01° x 0.01° | Reference dataset to improve upon | Rainfall dataset sourced from gauge data. SI is used to assimilate gauge data onto a background field of interpolated station climatological values. |
| GSMaP-NRT-V6, JAXA | 0.1° x 0.1° | Background field for the SI datasets in this study | Rainfall dataset sourced from satellite microwave and infrared data. Empirical relationships are used to convert microwave data to a rain rate, while IR data is used to increase spatiotemporal coverage. |
| ERA5, ECMWF | 0.1° x 0.1° | Reference dataset for the TCA validation | Rainfall data is sourced from a reanalysis produced using 4D-Var assimilation of observational data (not including rainfall from rain gauges) into the ECMWF Integrated Forecast System (IFS) |
| SM2R, Brocca et al.^30^ | 0.1° x 0.1° | Reference dataset for the TCA validation | Rainfall dataset derived from soil moisture estimates using empirical relationships. |
| MSWEP, H2O^26^ | 0.1° x 0.1° | Reference dataset for the gridded validation | Rainfall dataset sourced primarily from a blend of gauge data, GSMaP, ERA5 and IMERG. |
| OK-total-IEV, authors | 0.1° x 0.1° | Reference blended satellite dataset to compare against the satellite-SI datasets | Rainfall dataset sourced from a blend of GSMaP, AGCD and gauge data. Blended using weights derived from the error variances of GSMaP and AGCD. |
| station-climo-SI, authors | 0.1° x 0.1° | Dataset generated and validated in this study | Rainfall dataset sourced from gauge data. Should be very similar to AGCD except code used was specific to this study rather than the operational code. |
| OK-total-SI, authors | 0.1° x 0.1° | Dataset generated and validated in this study | Rainfall dataset sourced from gauge data and GSMaP. SI was used to assimilate gauge data onto a background field of corrected GSMaP. GSMaP was corrected using linear ratios of GSMaP totals to AGCD totals at station locations converted to a grid via Ordinary Kriging.^16^ |
| OK-anom-SI, authors | 0.1° x 0.1° | Dataset generated and validated in this study | Rainfall dataset sourced from gauge data and GSMaP. SI was used to assimilate gauge data onto a background field of corrected GSMaP. GSMaP was corrected using linear ratios of GSMaP anomalies to AGCD anomalies at station locations converted to a grid via Ordinary Kriging.^16^ |
| CDF-SI, authors | 0.1° x 0.1° | Dataset generated and validated in this study | Rainfall dataset sourced from gauge data and GSMaP. SI was used to assimilate gauge data onto a background field of corrected GSMaP. GSMaP was corrected using quantile to quantile matching of GSMaP to AGCD^16^ |
| ERA5-SI, authors | 0.1° x 0.1° | Dataset generated and validated in this study | Rainfall dataset sourced from gauge data and ERA5. SI was used to assimilate gauge data onto a background field of ERA5. |
